# A case of a shrunken multilocular mediastinal cyst that developed into thymic carcinoma with lung metastases 13 years later

**DOI:** 10.1111/1759-7714.15174

**Published:** 2023-11-28

**Authors:** Kazuto Sugai, Kojiro Nakaoka, Rika Tobita, Shinji Kikuchi, Kei Inoue, Midori Enokido, Moriyuki Kiyoshima

**Affiliations:** ^1^ Department of Thoracic Surgery Ibaraki Prefectural Central Hospital Kasama Japan; ^2^ Department of Thoracic Surgery University of Tsukuba Tsukuba Japan; ^3^ Department of Diagnostic Radiology Ibaraki Prefectural Central Hospital Kasama Japan

**Keywords:** mediastinal cyst, multilocular thymic cyst, thymic cancer

## Abstract

Multilocular thymic cysts (MTC) are acquired multilocular cysts caused by inflammation. The rarity of such lesions and a lack of recognition make diagnosis and treatment difficult. Herein, we present our experience with a multilocular mediastinal cyst that resulted in the development of thymic cancer with metastasis over a period of 13 years. Computed tomography findings revealed an anterior mediastinal mass that was suspected to be an MTC in a 49‐year‐old man. The mass shrank gradually over a period of 7 years; however, growth was observed at 10 years after initial detection. At 13 years after detection, thymic carcinoma with multiple lung metastases was diagnosed. Resection was recommended during the follow‐up period, but the patient refused treatment. A multilocular wall and location are factors that indicate MTC. However, even if a definitive diagnosis is not made, resection of multilocular anterior mediastinal cysts should be considered as determining the preoperative diagnosis is difficult. Nevertheless, our case suggests that the coexistence of tumors with cysts is possible, and the potential for malignant tumor development exists.

## Graphical abstract

A multilocular mediastinal cyst suspected as multilocular thymic cyst shrank over a period of 7 years; however, thymic cancer with lung metastasis developed 13 years after initial detection. Resection should be considered for multilocular mediastinal cysts, due to the challenges they pose in preoperative diagnosis, the potential for coexisting tumors with cysts, and the risk of malignant tumor development.

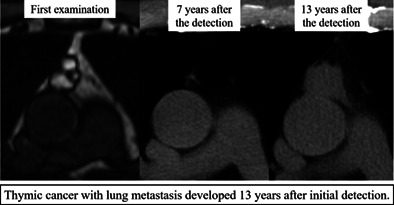

## INTRODUCTION

Thymic cysts can be categorized as congenital unilocular and acquired multilocular cysts that occur after inflammation. Although the removal of multilocular thymic cysts (MTCs) is recommended,^1^ their rarity and insufficient recognition make diagnosis and treatment difficult. Here, we present the case of a patient with a multilocular mediastinal cyst that was suspected to be an MTC. The patient was followed‐up for 13 years; however, during the follow‐up period, thymic cancer with multiple lung metastases developed. Thus, we present the natural history of a cyst suspected to be an MTC and recommend resection of multilocular anterior mediastinal cysts, including MTCs.

## CASE REPORT

**FIGURE 1:**
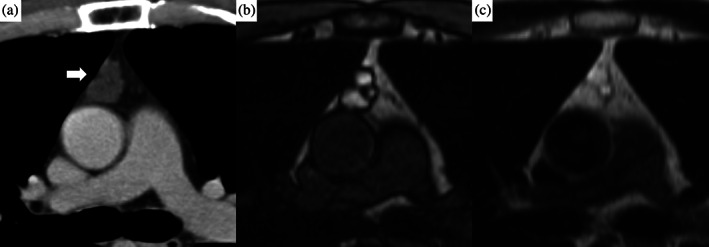
Anterior mediastinal images obtained during the initial examination. (a) Computed tomography imaging with a contrast agent. A 23‐mm, well‐defined, lobular mass was detected (white arrow). (b, c) High signal intensity was detected during T1‐ and T2‐weighted imaging. Because a multilocular wall was detected, the mass was considered to be a multilocular thymic cyst.

**FIGURE 2:**
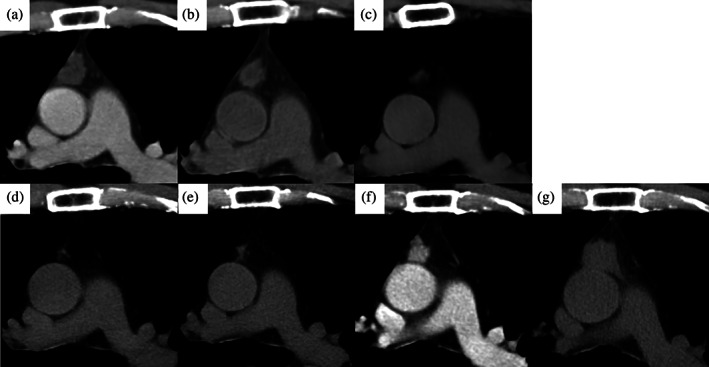
Anterior mediastinal computed tomography images of the mass. Images at (a) 3 years, (b) 4 years, and (c) 7 years after the mass was detected, showing that it had shrunk (d). Images at (e) 10 years, (f) 12 years, and (g) 13 years after the mass was detected, showing that it had grown.

**FIGURE 3:**
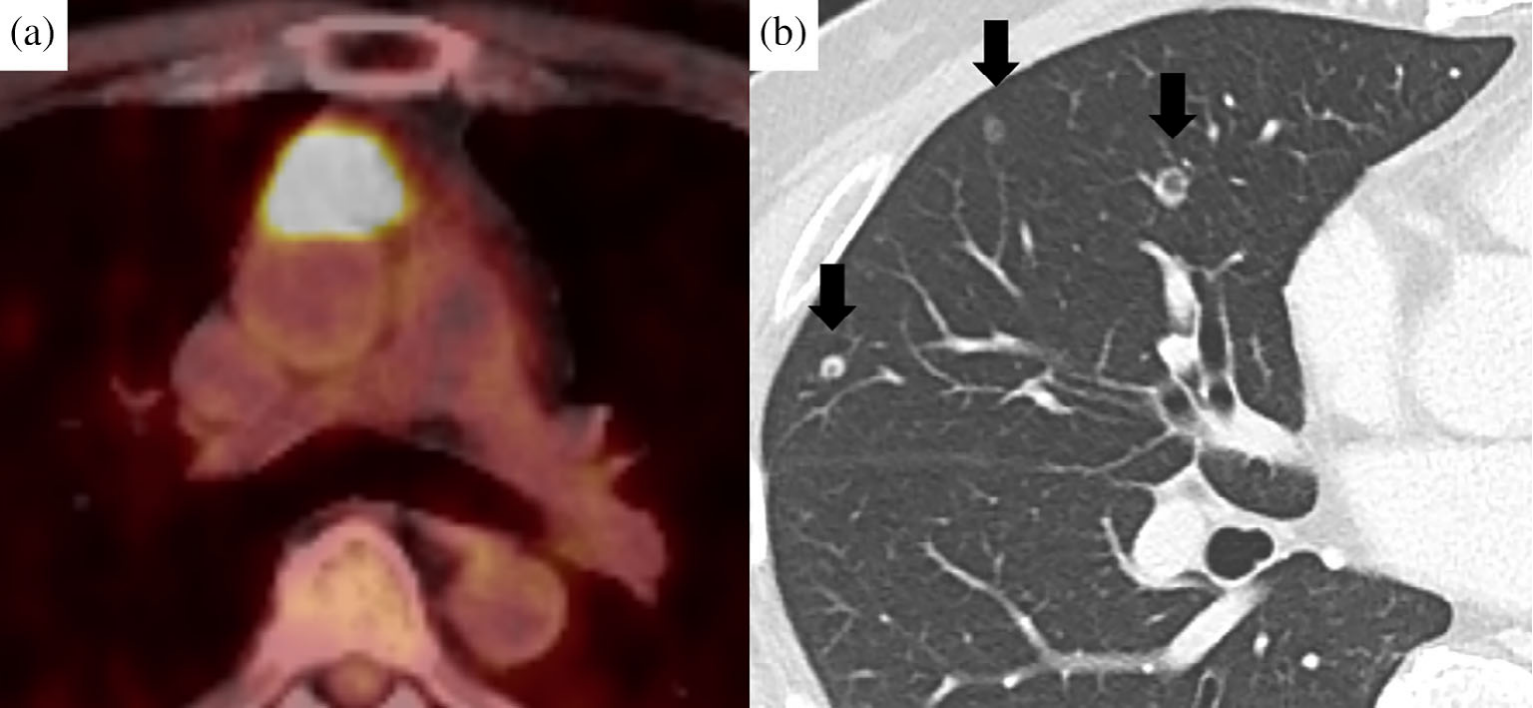
Images at 13 years after the initial examination. (a) Positron emission tomography‐computed tomography imaging showing strong accumulation of fluorodeoxyglucose in the anterior mediastinal tumor. (b) The bilateral lung nodules were suspected to be metastatic.

**FIGURE 4:**
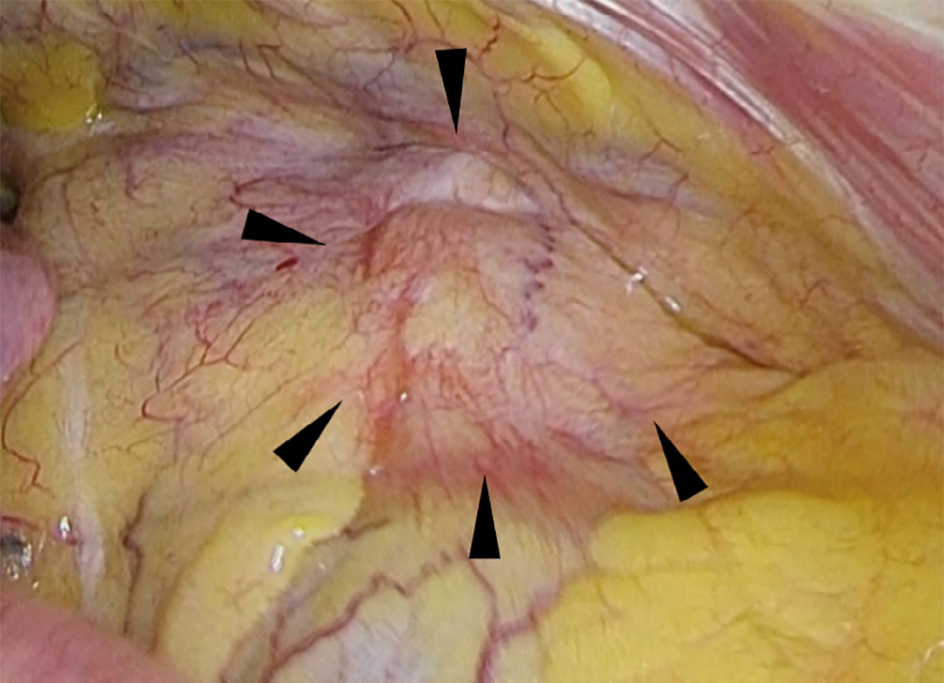
Image of intraoperative findings. The mediastinal mass (arrowhead) was strongly adherent to the pericardium, pericardial fat, and thymus.

An anterior mediastinal nodule was detected during computed tomography (CT) imaging of a 49‐year‐old man who was a non‐smoker and had a history of sudden deafness. During the first examination, the patient was asymptomatic, and laboratory findings were unremarkable. The CT images revealed a 23‐mm anterior mediastinal mass with irregular margins and clear borders without a solid region. Magnetic resonance imaging (MRI) revealed high T1 and T2 intensity and a multilocular wall (Figure 1). The mass was suspected to be an MTC, and resection was suggested. However, the patient refused; instead, he decided to undergo follow‐up. CT images obtained 1 year after the initial examination showed no growth. Positron emission tomography (PET)‐CT was performed during the follow‐up period; however, the mass did not show fluorodeoxyglucose (FDG) uptake. CT images obtained 3 and 4 years after the initial examination showed that the mass had gradually shrunk. Further, CT images taken 7 years after the first examination revealed that the mass had almost disappeared, with only a slight scar remaining. Although the mass was regarded as stable, CT imaging at 10 years after detection showed that it had slightly grown. CT imaging at 12 years after detection performed to screen for prostate cancer showed that the mass had increased to 17 mm. However, treatment for prostate cancer was prioritized. CT imaging at 13 years after detection revealed that the mass had grown to 29 mm (Figure 2), and was accompanied by multiple bilateral lung nodules (3–7 mm). PET‐CT subsequently revealed strong FDG uptake with maximum standardized uptake values of 29.5 (1 h) and 33.3 (2 h) (Figure 3). The anterior mediastinal mass was suspected to be thymic cancer with multiple lung metastases, and a biopsy of the mediastinal mass and lung nodule was planned. Rapid pathological evaluation of tissue obtained by initial wedge resection of the lung nodule revealed squamous cell carcinoma. Given this finding, thymic cancer with lung metastases was considered likely. Complete resection of the thymic cancer was impossible due to metastases; we therefore planned a less invasive and shorter surgery followed by subsequent chemotherapy. The mediastinal mass was barely identifiable due to the strong adhesion to the pericardium, pericardial fat, and thymus (Figure 4). Surgery was completed after an incisional biopsy of a 5 cm region of the mediastinal area following pulmonary biopsy. Pathological examination results revealed that the majority of the mediastinal specimen had fat tissues, with some intravascular tumor clusters. Both the pulmonary and mediastinal specimens were c‐kit‐positive and CD5‐positive, and the patient was finally diagnosed with thymic cancer with multiple lung metastases. Fibrotic regions were identified in the mediastinal specimen, but the diagnosis of MTC was challenging to make. Chemotherapy comprising carboplatin and nab‐paclitaxel was administered.

## DISCUSSION

During follow‐up of this case of a suspected MTC, the mass shrank. However, at 10 years after detection, growth was observed, ultimately resulting in the diagnosis of thymic carcinoma with multiple lung metastases at 13 years after its detection. Resection was suggested several times based on prior results indicating that MTC had a risk of developing into malignancy.^1^, ^2^ However, the patient refused. As we could not predict the outcome, we could not strongly recommend resection for this patient.

Thymic cysts account for 3%–5% of mediastinal tumors,^2^, ^3^ and are classified as monocular or multilocular based on their internal morphology. Most cases are monocular, and MTCs are relatively rare. In 1991, Suster et al. reported that MTCs are defined by the following four characteristics: multiple cystic cavities with squamous, columnar, or cuboidal epithelium; islands of non‐neoplastic thymic tissue in the cyst walls; strong inflammatory changes; and lymphatic hyperplasia.^4^, ^5^ Although the exact etiology is unknown, MTCs are known to be caused by inflammatory processes,^4^ including Sjögren's syndrome,^1^, ^3^ radiation,^6^ and surgical trauma.^7^ In this case, the mediastinal multilocular cyst had already shrunk prior to surgery, and the resected mediastinal specimen contained only few tumor cells, which limited the ability to confirm the pathological diagnosis of MTC based on Suster's report.^4^ Only fibrosis indicated an inflammatory process, but this was insufficient for a complete diagnosis of MTC. Further, pathological diagnosis requires resection of the cyst; however, this prevents the evaluation of the tumor's natural history. Therefore, this case report holds value, even though some aspects of the pathological features may be inconclusive.

The histopathological features of MTC have previously been described in the literature, but the standard imaging findings of MTC have not yet been established.^3^, ^4^ In the present case, a pathological diagnosis was not achieved, and imaging evaluation of the anterior‐mediastinal cyst was important. Differential diagnoses on imaging of MTC include benign diseases: pericardial cysts, cystic teratomas, lymphangiomas, hemangiomas, and cystic degeneration of malignant diseases, including seminomas and Hodgkin's diseases. A septum within the cyst is a unique finding for distinguishing MTC among these diseases. However, with imaging alone, it is challenging to confidently differentiate MTC from these other potential diagnoses. Teratomas often show features such as fat, cartilaginous tissue, or tooth‐like calcification, and have a thick wall. Lymphangiomas typically contain cervical or axillary components. Hemangiomas exhibit contrast enhancement. Cystic degeneration of malignancies often presents as cysts with solid components. Additionally, these differential diagnoses typically do not demonstrate spontaneous shrinkage, except for unilocular cysts. Therefore, the multilocular mediastinal cyst in this case was suspected to most likely be MTC based on imaging and clinical course.

Resection is recommended for MTCs as preoperative diagnosis is difficult and some tumors to be undetectable during imaging.^1^, ^2^ Their rarity and lack of recognition make their diagnosis and treatment difficult. Additionally, the natural course of MTCs has not yet been reported as this condition is rare and poorly recognized, and resection is generally performed. The mechanism underlying cyst development and the reason for its spontaneous regression were unknown in this case. It is unclear whether a thymic tumor coexisted with the MTC at the time of the initial diagnosis, whether new thymic cancer developed in the background of the MTC, or whether the thymic carcinoma developed at the site of intense inflammation that led to MTC. Regardless, the mediastinal multilocular cyst suspected as MTC resulted in thymic cancer in the present case. In conclusion, we advocate for clinicians to consider resection in cases of multilocular anterior mediastinal cysts, including MTCs, due to the challenges they pose in preoperative diagnosis, the potential for coexisting tumors with cysts, and the risk of malignant tumor development.

## AUTHOR CONTRIBUTIONS

All authors have read and take responsibility for the manuscript. Kazuto Sugai: Conceptualization, visualization, writing–original draft preparation, writing–review, and editing. Kojiro Nakaoka, Shinji Kikuchi, Moriyuki Kiyoshima: Data curation. Kei Inoue and Midori Enokido: Writing–review and editing. Moriyuki Kiyoshima: Supervision.

## CONFLICT OF INTEREST STATEMENT

The authors have no conflict of interest.

## Data Availability

The data supporting the findings of this case are available from the corresponding author upon reasonable request.
